# Role of Pelvic Lymph Node Resection in Vulvar Squamous Cell Cancer: A Subset Analysis of the AGO-CaRE-1 Study

**DOI:** 10.1245/s10434-021-09744-y

**Published:** 2021-03-15

**Authors:** Linn Woelber, Mareike Bommert, Philipp Harter, Katharina Prieske, Christine zu Eulenburg, Julia Jueckstock, Felix Hilpert, Nikolaus de Gregorio, Severine Iborra, Jalid Sehouli, Atanas Ignatov, Peter Hillemanns, Sophie Fuerst, Hans-Georg Strauss, Klaus Baumann, Matthias Beckmann, Alexander Mustea, Werner Meier, Sven Mahner, Anna Jaeger

**Affiliations:** 1 Department of Gynecology and Gynecologic Oncology, University Medical Center Hamburg–Eppendorf, Hamburg, Germany; 2 Department of Gynecology and Gynecologic Oncology, Evang. Kliniken Essen-Mitte, Essen, Germany; 3 Mildred Scheel Cancer Career Center HaTriCS4, University Medical Center Hamburg–Eppendorf, Hamburg, Germany; 4 Department of Epidemiology, UMCG, Universität Groningen, Groningen, The Netherlands; 5 Department of Obstetrics and Gynecology, University Hospital, LMU – University of Munich, Munich, Germany; 6 Oncologic Medical Center at the Jerusalem Hospital Hamburg, Hamburg, Germany; 7 Department of Obstetrics and Gynecology, University of Ulm Medical Center, Ulm, Germany; 8 Department of Gynecology and Gynecologic Oncology, University Hospital Aachen, RWTH Aachen, Aachen, Germany; 9 Department of Gynecology, Charité University Medicine Berlin, Campus Virchow, Berlin, Germany; 10 Department of Obstetrics and Gynecology, University Hospital Magdeburg, Magdeburg, Germany; 11 Department of Obstetrics and Gynecology, Hannover Medical School, Hannover, Germany; 12 Department of Gynecology, University Hospital Halle, Halle, Germany; 13 Department of Gynecology, Medical Center Ludwigshafen, Ludwigshafen, Germany; 14 Department of Gynecology and Obstetrics, University Hospital Erlangen, Comprehensive Cancer Center Erlangen-EMN, Friedrich-Alexander University Erlangen-Nuremberg, Erlangen, Germany; 15 Department of Gynecology and Gynecologic Oncology, University Medical Center Bonn, Bonn, Germany; 16 Department of Obstetrics and Gynecology, Evangelical Hospital Duesseldorf, Duesseldorf, Germany

## Abstract

**Background:**

As the population at risk for pelvic nodal involvement remains poorly described, the role of pelvic lymphadenectomy (LAE) in vulvar squamous cell cancer (VSCC) has been a matter of discussion for decades.

**Methods:**

In the AGO-CaRE-1 study, 1618 patients with International Federation of Gynecology and Obstetrics (FIGO) stage IB or higher primary VSCC treated at 29 centers in Germany between 1998 and 2008 were documented. In this analysis, only patients with pelvic LAE (*n* = 70) were analyzed with regard to prognosis and correlation between inguinal and pelvic lymph node involvement.

**Results:**

The majority of patients had T1b/T2 tumors (*n* = 47; 67.1%), with a median diameter of 40 mm (2–240 mm); 54/70 patients (77.1%) who received pelvic LAE had positive groin nodes. For 42 of these 54 patients, the number of affected groin nodes had been documented as a median of 3; 14/42 (33.3%) of these patients had histologically confirmed pelvic nodal metastases (median number of affected pelvic nodes 3 [1–12]). In these 14 patients, the median number of affected groin nodes was 7 (1–30), with a groin metastases median maximum diameter of 42.5 mm (12–50). Receiver operating characteristic analysis showed an area under the curve of 0.85, with 83.3% sensitivity and 92.6% specificity for the prediction of pelvic involvement in cases of six or more positive groin nodes. No cases of pelvic nodal involvement without groin metastases were observed. Prognosis in cases of pelvic metastasis was poor, with a median progression-free survival of only 12.5 months.

**Conclusion:**

For the majority of node-positive patients with VSCC, pelvic nodal staging appears unnecessary since a relevant risk for pelvic nodal involvement only seems to be present in highly node-positive disease.

Pelvic nodal involvement in primary vulvar squamous cell cancer (VSCC) is considered rare, and it is estimated that <10% of all VSCCs and <2% of early VSCCs show nodal spread beyond the groin to the pelvis.^1^–^4^ The question of when and if pelvic lymphadenectomy (LAE) should be performed in VSCC has been the subject of discussion since the 1980s. The Gynecologic Oncology Group (GOG) addressed the question in a randomized trial (GOG37) by Homesley et al.^5^ Patients with histologically confirmed inguinal lymph node metastases after surgical groin dissection received either 45–50 Gy of radiotherapy to both the groin and the pelvis, or pelvic LAE without any adjuvant radiotherapy. In 15/53 patients (28.3%) in the pelvic LAE group, histologically confirmed positive pelvic nodes were detected. The GOG37 study was closed earlier than planned due to a significant survival benefit in the radiotherapy group. Since the publication of these study results, adjuvant radiotherapy of the groin and pelvis has been implemented as the standard therapeutic approach to VSCC with more than one lymph node metastasis in the groin; however, due to the design of the study, interpretation of the results remains difficult. Two-year overall survival (OS) was superior in the radiotherapy group compared with the pelvic LAE group’ (68% vs. 54%), while the pelvic recurrence rate was higher in the ‘radiotherapy group’ (6% vs. 2%). The poor outcome of the pelvic LAE group can mainly be attributed to the omission of adjuvant radiotherapy to the groin in this group, resulting in a higher groin recurrence rate of 23.6%, compared with only 5.1% in the radiotherapy group, as the prognosis of groin recurrences is known to be fatal in the majority of cases, with a 5-year OS rate of only 20%.^6^ The named study could therefore not clarify the role of pelvic LAE in node-positive VSCC.

As the overall risk for pelvic nodal involvement is estimated to be 20–35% in node-positive VSCC and radiotherapy of the pelvis can cause substantial morbidity, especially in the elderly population affected by VSCC, it is currently recommended by some treatment guidelines to perform systematic pelvic LAE as a staging procedure (by the minimally invasive or retroperitoneal approaches) in patients at risk for pelvic nodal involvement;^7^ however, this approach often requires secondary surgery with increased morbidity and the population at risk is poorly defined. In a pilot study from Charité Berlin, 12 patients with node-positive primary or recurrent VSCC (1–7 affected groin nodes) received pelvic LAE, however only two of these patients showed pelvic nodal involvement (17%).^3^ The risk of pelvic nodal metastases appears to increase with the number of affected groin nodes.^8^,^9^ According to the German guideline, patients at risk for pelvic metastases include patients with one groin metastasis >5 mm, patients with two or more groin metastases (including bilateral involvement), and metastases with extracapsular spread.^7^ These characteristics are known to be associated with poor prognosis in general. The role of pelvic nodal involvement in this subgroup and its impact on prognosis is still unclear. Furthermore, the questions as to when and how to perform pelvic LAE, as well as the optimal extent of pelvic treatment generally in patients with node-positive VSCC, is still surrounded by considerable controversy. Therefore, the aim of the current study was to analyze the relation between lymph node involvement of the groin and pelvis and the relevance of pelvic metastasis for prognosis based on all patients who were treated with pelvic LAE within the Arbeitsgemeinschaft Gynäkologische Onkologie–Chemo and Radiotherapy in Epithelial Vulvar Cancer-1 (AGO-CaRE-1) study.

## Methods

The current analysis evaluated a subgroup of the AGO-CaRE-1 study.^10^ The aim of the AGO-CaRE-1 study, a large retrospective study, was to survey treatment patterns as well as prognostic factors in VSCC. Overall, 1618 patients with International Federation of Gynecology and Obstetrics (FIGO) stage IB or higher primary VSCC (Union for International Cancer control [UICC] TNM classification and stage groupings version 6) treated at 29 gynecologic cancer centers in Germany between 1998 and 2008 were included.^11^ Participating institutions were asked to include all patients with a diagnosis of stage >pT1a invasive vulvar cancer, independent of the mode and initial place of treatment. Data collection was performed retrospectively between February and December 2011. Documentation and analysis were undertaken by the AGO study group through a specifically designed centralized database. The study was approved by each local Ethics Committee (leading vote: Hamburg, reference number PV3658, and registered with ClinialTrials.gov [NCT01304667]). The results of the main analysis have been previously published.^10^ This subset analysis is a retrospective data collection study that focuses on patients with pelvic LAE (*n* = 70), and evaluates, in particular, the occurrence of pelvic nodal involvement at primary diagnosis as well as the correlation between inguinal and pelvic nodal involvement. Furthermore, the impact of pelvic nodal metastases on prognosis was investigated.

### Statistical Analysis

Analysis was performed using Stata version 14.2 (StataCorp LLC, College Station, TX, USA). Variables are described as median and range or count and percentage. Receiver operating characteristics (ROC) analysis was performed and the area under the curve (AUC) was calculated to evaluate different cut-offs for the prediction of pelvic nodal involvement related to the number of affected groin nodes. Progression-free survival (PFS) was calculated as the time interval between primary diagnosis and disease progression or death of any cause, while OS was defined as the period from primary diagnosis to death of any cause. Univariate Cox regression analysis was applied to determine significant differences at a level of 5%.

## Results

### Patients

Of 1618 patients with stage IB–IV VSCC treated between 1998 and 2008 at one of the 29 participating German cancer centers, only 70 patients received surgical staging of the pelvis (pelvic LAE) and had a known lymph node status of the groin (*n* = 54 node-positive; *n* = 16 node-negative). Patient characteristics are displayed in Table 1. Median age was 63 years (range 20–85) and median follow-up was 31 months (range 1–140). The majority of patients had locally restricted tumors (T1b/T2; TNM staging system version 6: 47/70, 67.1%), which were predominantly resected with tumor-free margins (41/57 R0, 71%).^12^

**Table 1:** Patient characteristics (*n* = 70) with regard to pelvic lymph node status

	Total [*N* = 70]	Pelvic status missing [*n* = 13]	Pelvic pN+ [*n* = 14]	Pelvic pN− [*n* = 43]	*p*-value (N+ vs. N−)
Age, years [median (range)]	63.0 (20.3–85.2)	68.1 (52.0–79.2)	71.5 (31.5–82.8)	56.2 (20.3–85.2)	0.026^a^
Tumor stage					
pT1b	12	4	3	5	0.604^b^
pT2	35	4	6	25	
pT3/4	1	0	0	1	
Unknown	22	5	5	12	
Nodal status					
pN0	16	2	0	14	0.010^b^
pN1	54	11	14	29	
No. of groin nodes affected [median (range)], *n* = 42	3 (1–30)	2 (2–2)	7 (1–30)	2 (1–10)	0.001^a^
Maximum diameter of LN metastases of the groin, mm (range), *n* = 9	27.0 (1–50)	10 (10–10)	42.5 (12–50)	23.5 (1–32)	0.187^a^
No. of pelvic nodes affected [median (range)], *n* = 14	2.5 (1–12)	NA	2.5 (1–12)	NA	NA
Tumor diameter, mm [median (range)], *n* = 53	40 (2–240)	40 (18–70)	40 (15–240)	39 (2–110)	0.106^a^
Depth of invasion, mm [median (range)], *n* = 26	5.7 (1.5–70)	3 (3–3)	5.3 (5–6)	9 (1.5–70)	0.357^a^
Grading					
G1	3	0	0	3	0.024^b^
G2	34	3	4	27	
G3	29	7	10	12	
Unknown	4	3	0	1	
ECOG					
0	23	1	0	22	<0.001^b^
1	9	0	4	5	
2	12	5	1	6	
3	3	0	3	0	
4	1	0	1	0	
Unknown	22	7	5	10	
Surgical therapy vulva					0.950^b^
Partial vulvectomy	19	2	3	14	
Complete vulvectomy	45	10	10	25	
Exenteration/unknown	6	1	1	4	
Resection margin, mm [median (range)], *n* = 19	4 (0.2–11)	4.75 (0.5–9)	3 (2–4)	4 (0.2–11)	0.496^a^
Resection status					
R0	41		9	32	0.443^b^
R1	12		3	9	
Rx	4		2	2	
Type of groin surgery					
Complete (groin dissection)	69	13	14	42	1.000^b^
Sentinel (only)	1	0	0	1	
Sentinel node biopsy performed					
No	47	5	12	30	0.024^b^
Yes	15	3	0	12	
Unknown	8	5	2	1	
No. of dissected LNs (groin) per patient [median (range)], *n* = 57	17 (2–53)	17 (5–37)	15 (6–36)	17 (2–53)	0.935^a^
No. of dissected LNs (pelvis) per patient [median (range)], *n* = 51	12 (1–55)	NA	10 (1–28)	13 (2–55)	0.761^a^
Radiotherapy performed					
Yes	38	9	10	19	0.171
No	24	2	2	20	
Unknown	8	2	2	4	
Type of radiotherapy					
Adjuvant therapy	33	8	8	17	0.213^b^
Neoadjuvant therapy	2	0	0	2	
Palliative	3	1	2	0	
Unknown	32	4	4	24	
Radiation fields					1.000^b^
Groin ± vulva	8	1	2	5	
Groin and pelvis ± vulva	21	5	6	10	
Pelvis ± vulva	5	2	1	2	
Neither groin nor pelvis	2	0	0	2	
Unknown	34	5	5	24	
Median PFS (months)	35.2	38.0	12.5	41.3	0.020^c^
Median OS (months)	NR	72.5	30.8	NR	0.003^c^

*ANOVA* analysis of variance, *LN* lymph node, *ECOG* Eastern Cooperative Oncology Group, *PFS* progression-free survival, *OS* overall survival, *NR* not reached, *n. a.* not applicable

^a^ANOVA

^b^Fisher’s exact test

^c^Cox regression analysis

‘Missing’ and ‘unknown’ categories excluded

**Fig. 1:**
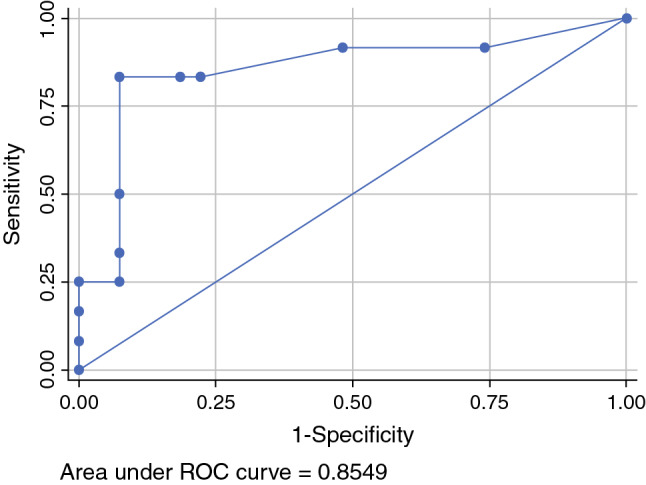
Prediction of pelvic involvement depending on the number of inguinally affected lymph nodes. *ROC* receiver operating characteristic

Overall, 54/70 patients with pelvic LAE (77.1%) showed positive inguinal nodes (N+). In the total cohort, pelvic nodal involvement without groin metastases was not observed. Information regarding the number of groin nodes affected was available in 42 node-positive patients (median number of nodes affected = 3); 14/42 (33%) patients showed both inguinal and pelvic metastases, with a median of 3 (range 1–12) affected pelvic lymph nodes. In the pelvic node-positive group, the median number of affected groin nodes was 7 (range 1–30), with a median metastatic diameter of 42.5 mm (range 12–50). Ten pelvic node-positive patients had six or more positive lymph nodes in the groin, while one patient had just one groin node metastasis (Table 2); unfortunately, the size of the groin metastasis was not available in this particular patient. ROC analysis showed an AUC of 0.85, with 83.3% sensitivity and 92.6% specificity for the prediction of pelvic involvement in cases of six or more positive groin nodes (Fig. 1).

**Table 2:** Relation between inguinal and pelvic nodal involvement (*n* = 70 patients with pelvic lymphadenectomy; *n* = 12 pelvic node-positive patients with known number of affected groin nodes)

No. of positive lymph nodes (groin)	No. of patients with negative pelvic LN status	No. of patients with positive pelvic LN status	Total
1	7	1	8
2	7	0	7
3	7	1	8
4	1	0	1
5	3	0	3
6	0	4	4
8	0	2	2
9	0	1	1
10	2	0	2
11	0	1	1
12	0	1	1
30	0	1	1
Total	27	12	39

A total of 42.9% (30/70) of patients experienced any kind of disease recurrence after a median of 9.2 months (range 1.5–73.1) [Table 3]. However, while taking into account the fact that the radiotherapy fields were not documented in all patients (see Table 1), 50% (7/14) and 28% (12/43) of the pelvic node-positive and pelvic node-negative patients, respectively, received radiotherapy including the pelvis. No pelvic recurrences were observed in the pelvic node positive group whereas pelvic recurrences occured in 7% (3/43 patients) within the pelvic node negative group. In the pelvic node-positive group, 28.6% (4/14) of patients experienced distant recurrences as the most frequent site, followed by local recurrences on the vulva in 21.4% (3/14) of patients. In the pelvic node-negative group, recurrences appeared most often on the vulva (10/43 patients, 23.3%), followed by the pelvis and distant metastases in 3/43 (7%) patients. Death before recurrence occurred in 11.4% (8/70) of patients after 21.1 months (range 1.31–45.83). As expected, the risk of recurrence was higher in the pelvic node-positive group compared with the pelvic node-negative group (8/14 patients [57.1%] vs. 17/43 patients [39.5%]). Within the 31-month follow-up, 51.2% (22/43) of node-negative patients versus 28.6% (4/14) of node-positive patients remained free of recurrences.

**Table 3:** Site of disease recurrence

Localization of disease recurrence	Total (*n* = 70 patients), 30 recurrences [42.9%]	Node-negative pelvis (*n* = 43 patients), 17 recurrences [39.5%]	Node-positive pelvis (*n* = 14 patients), 8 recurrences (57.1%)	Pelvic status unknown (*n* = 13), 5 recurrences (38.5%)
No recurrence	32 (45.7)	22 (51.2)	4 (28.6)	6 (46.2)
Vulva only	15 (21.4)	10 (23.3)	3 (21.4)	2 (15.4)
Groin only	1 (1.4)	1 (2.3)	0	0
Vulva + groin	3 (4.3)	0	1 (7.1)	2 (15.4)
Pelvis (± other localizations)	4 (5.7)	3 (7)	0	1 (7.7)
Distant (± other localizations)	7 (10)	3 (7)	4 (28.6)	0
Death before recurrence	8 (11.4)	4 (9.3)	2 (14.3)	2 (15.4)

Data are expressed as *n* (%)

**Fig. 2:**
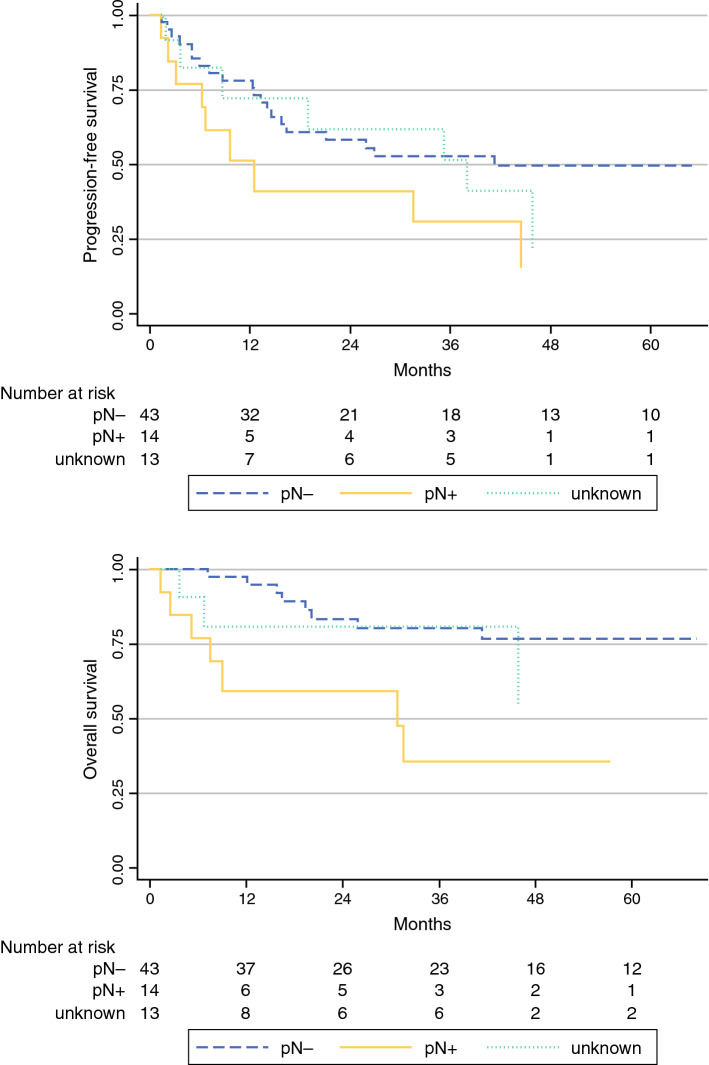
Progression-free survival and overall survival with regard to pelvic lymph node status

The median PFS for all patients, regardless of pelvic node status, was 35.2 months, while the median OS was not reached. In the case of pelvic metastasis, prognosis was significantly impaired, with a median PFS of only 12.5 months and a median OS of 30.8 months (Fig. 2).

### Discussion

When and how to treat the pelvis in patients with node-positive VSCC is still an open question. Pelvic LAE as a staging procedure in patients with inguinal node-positive VSCC is subject to controversial discussion in many countries due to simultaneously performed irradiation of the groin *and* pelvis in the detection of groin metastases. This practice was implemented after publication of the GOG37 study in the 1980s, however this study was not designed to answer the question, ‘who actually benefits from pelvic treatment at all (radiotherapy and/or LAE)? The results of the current study confirm that pelvic nodal involvement can be expected in approximately 30% of all patients with positive groin nodes, which is in line with previously reported rates, namely the 28% described by Homesley et al.^5^ (15/53 patients). However, negative selection bias in the current analysis is likely, as the indication for pelvic LAE was individually posed, and a relative overestimation of pelvic involvement with regard to all node-positive patients cannot be excluded. Nevertheless, this also means that approximately 70% of all node-positive patients likely do not need any kind of pelvic treatment or any surgical staging.

The main limitations of our data were that the data were generated in a period when preoperative radiologic staging was not routinely implemented as standard in all patients with locally advanced vulvar cancer, and that this was a post hoc analysis. Therefore, we missed the collection of data and/or scans of radiologic imaging. We know the limitation of imaging regarding inguinal nodes, but this is completely unknown for the question we addressed in this work.

Overall, the relatively low incidence of positive pelvic nodes, the increased surgical morbidity with potentially delayed adjuvant radiotherapy, and the generally poor prognosis of node-positive patients make it questionable as to whether there is sufficient justification for pelvic LAE as a staging procedure. Nonetheless, a subset of patients at high risk for pelvic nodal involvement may benefit from systematic pelvic LAE, when adjuvant radiotherapy to the pelvis can be avoided in the case of negative pelvic nodes. This could be especially relevant for younger women with open family planning or patients with comorbidities complicating radiotherapy.

VSCC-related recurrence and death rates are consistently predicted by nodal involvement as inguinal lymph node metastases continue to be the most important prognostic factor for both PFS and OS (3-year PFS and OS rates of 35.2% and 56.2%, respectively, in node-positive patients versus 75.2% and 90.2%, respectively, in node-negative patients).^3^,^5^,^10^,^13^,^14^ The number of affected groin nodes thereby correlates with survival (27% 2-year OS for patients with four or more positive groin nodes, 66% 2-year OS for patients with two to three positive groin nodes, and 88% 2-year OS for patients with only one positive groin node; *p* < 0.0001).^5^,^15^ One possible reason for this relation might be the increasing risk for pelvic nodal involvement with the increasing number of groin nodes affected. Furthermore, the risk for pelvic metastases seems to be only relevant in highly groin node-positive disease. However, the prediction of pelvic nodal involvement in view of inguinal metastases with a clinically sufficient specificity remains an unresolved issue.

Hacker et al. were one of the first to determine pelvic nodal involvement in patients with positive groin nodes.^8^ In their study, neither initial pelvic involvement nor pelvic recurrence were observed in cases of two or fewer positive groin nodes, while 2/3 patients (66.6%) with three positive groin nodes and 5/6 patients (83.3%) with four or more positive groin nodes experienced pelvic nodal involvement. Irrespective thereof, no patients with confirmed pelvic metastasis were cured of the disease. An increased risk for pelvic involvement in patients with three or more positive groin nodes was also observed in other small cohorts.^8^,^16^ In our cohort, a clinically valid prediction of pelvic involvement could only be made in cases of six or more positive groin nodes. Unfortunately, information regarding certain decisive factors with potential influence on the description of the relation between inguinal and pelvic nodes, such as laterality of the pelvic metastases, was not documented in the CaRE-1 database.

Of note, a relevant proportion of patients in the investigated cohort were receiving pelvic LAE despite histologically negative groin nodes (>20%). The reasons for this can only be speculated on, but reactive enlargement of groin nodes clinically suspicious for metastatic disease might be the main factor. It has been described that in up to 30% of clinical examinations, groin nodes are categorized as suspicious, although histology later does not show metastases.^8^ Furthermore, the role of imaging in early-stage VSCC remains debatable. The accuracy of sonography ranges between 67 and 89%,^17^ the sensitivity of magnetic resonance imaging is 89% with accuracy of 90%^18^ and the sensitivity of positron emission tomography (PET) is only 80% for the detection of lymph node metastases.^19^ Thus, imaging will not solve the problem of prediction of pelvic lymph node involvement in most cases.^20^ Simultaneous pelvic LAE without previous confirmation of groin metastases should therefore be avoided.

In accordance with the previous data, pelvic nodal involvement without groin metastases was not observed in our study. Another notable finding from our analysis is that while 28.6% of pelvic node-positive patients experienced recurrences at distant sites (4/14), no pelvic recurrences were observed in the pelvic node-positive subgroup (in contrast to the pelvic node-negative group, in which a 7% pelvic recurrence rate was observed). One could now speculate that this is an effect of adjuvant pelvic radiotherapy in the node-positive group or a lack of surgical radicality during LAE in the node-negative group. Furthermore, the omittance of pelvic radiotherapy in the case of negative pelvic staging could possibly increase the risk of pelvic recurrence in the node-negative group. In this context, Curry et al. also reported a pelvic rate of recurrence in at least 8% in patients who had fewer than four positive inguinal lymph nodes and whose pelvic lymph nodes were initially node-negative.^9^ However, Homesley et al. reported a slightly lower rate of pelvic recurrence of 4.4% (5/114 patients) in their total patient population and 1.8% (1/55 patients) in the cohort treated with pelvic LAE.^5^ Taken together, pelvic LAE might have been of beneficial impact, or rather the omittance of pelvic radiotherapy in the pelvic node-negative group might have increased the risk for pelvic recurrences. Data regarding the effectiveness of chemotherapy in pelvic node-positive patients and patients with distant/pelvic recurrences are even more sparse. The most commonly used agents are paclitaxel, bleomycin, cisplatin, and 5-fluorouracil; however, numbers are too small to draw a conclusion.

Although the CaRE-1 study represents one of the largest VSCC cohorts, our results show that only a small number of patients receive pelvic LAE (70/1618, 4.3%), including a considerably high rate of inguinal node-negative patients (16/70, 22.9%). A future goal is to precisely predict who is at risk for pelvic involvement while simultaneously preventing overtreatment and unnecessary harm to a majority of mostly older and comorbid patients.

The current study shows that only patients with a high disease burden in the groin seem to be at danger, but the numbers are to small to put this into clinical practice. Therefore, our team along with the German AGO study group have already received permission from the Ethics Committee to commence a multicenter trial of 35 centers in Germany (AGO–VOP.2) in order to evaluate the relation of inguinal and pelvic nodal metastases and the optimal treatment approach.

## Conclusion

In view of the poor prognosis, the relatively low incidence of pelvic nodal involvement, and the associated increase in surgery-related morbidity, surgical staging of the pelvis appears unnecessary for the majority of patients with inguinal node-positive VSCC. However, in a subset of patients at high risk for pelvic nodal involvement, pelvic LAE might represent an alternative approach for omitting radiotherapy of the pelvis in cases of negative pelvic lymph node status, e.g. in younger patients with open family planning. Further systematic data, as planned by the German AGO study group, the AGO Kommission Vulva Vagina, and the Nord-Ostdeutsche Gesellschaft für Gynäkologische Onkologie (NOGGO) is needed to further investigate the indication criteria for pelvic LAE, as well as the impact of the latter on the prognosis and outcome of affected patients.
